# Universal health coverage and medical industry in 3 Southeast Asian countries

**DOI:** 10.1186/1471-2458-14-S1-I3

**Published:** 2014-01-29

**Authors:** Laksono Trisnantoro

**Affiliations:** 1 Universitas Gadjah Mada, Yogyakarta 55281, Indonesia

## 

Indonesia, Malaysia, and Thailand experienced changes in government expenditure in health. Indonesia and Thailand, move to more public financing. These countries did not have history of universal coverage and the governments have political motive for universal coverage. The case of Malaysia works in opposite direction: some members of communities (the affluent ones) are not satisfied with certain services and demand better health service using private financing. The policy issues is: how does the government policy for achieving universal health care also manage health service as an industry? This analysis of the issue shows: (1) universal coverage will have pressure for government fiscal condition; (2) private medical service will be “a good safety valve” in reducing the burden of public finance for health; (3) medical industry policy should support the development of private medical services but considering equity issues. The impact of universal coverage and medical industry policies are: more segmented hospitals based on technology and economy status; more diverse sources of health financing (public and private); more mechanisms of funding: fee-for-service, indemnity in commercial health insurance, managed care, and others. These impacts need a carefully crafted health policy within the broader social and economic/industrial policy.

